# Functional versus mechanical alignment in robotic primary total knee arthroplasty: A meta-analysis

**DOI:** 10.1051/sicotj/2026061

**Published:** 2026-09-01

**Authors:** Youssef Jamaleddine, Ralph Maroun, Chahine Assi, Alfred Khoury, Pascal Kouyoumdjian

**Affiliations:** 1 Department of Orthopedic Surgery, Lebanese American University Medical Center-Rizk Hospital Beirut Lebanon; 2 Department of Orthopedic Surgery, Centre Hospitalier Universitaire de Nîmes Nîmes France

**Keywords:** Robotic, Total knee arthroplasty, Functional alignment, Mechanical alignment, Outcome

## Abstract

*Introduction*: Functional alignment (FA) has emerged as a robot-enabled, patient-specific strategy in total knee arthroplasty (TKA), aiming to individualize component positioning and knee balance within predefined safety limits. However, whether robotic FA provides clinically meaningful advantages over robotic mechanical alignment (MA) remains uncertain. This meta-analysis compared robotic FA versus robotic MA in primary TKA. *Methods*: Medline, the Cochrane Library, and Google Scholar were searched from inception to December 29, 2025, for comparative studies evaluating FA versus MA in robotic primary TKA. Outcomes included additional soft-tissue release, reported knee motion, Forgotten Joint Score-12 (FJS-12), 1-year functional score improvement using Oxford Knee Score or converted Western Ontario and McMaster Universities Osteoarthritis Index scores, and operative duration. Continuous outcomes were pooled as mean differences (MDs), and dichotomous outcomes as odds ratios (ORs). *Results*: Six studies including 645 TKAs were included. MA was associated with higher odds of requiring additional soft-tissue release compared with FA (OR = 5.38; 95% CI: 2.71 to 10.69; *P* < 0.00001). FA showed greater early improvement in reported knee motion at ≤6 weeks (MD = −4.64°; 95% CI: −7.07 to −2.21; *P* = 0.0002), but this difference was no longer significant at 6 months (MD = −1.13°; 95% CI: −8.24 to 5.98; *P* = 0.76). FJS-12 scores were similar at 3 months but favored FA at 6 months (MD = −10.74; 95% CI: −13.65 to −7.83; *P* < 0.00001), although this did not reach the established threshold for clinical importance. Functional score improvement at 1 year was similar between groups. Operative time statistically favored FA, although the difference was small. *Conclusion*: Robotic FA was associated with fewer soft-tissue releases and greater early improvement in reported knee motion, but these early advantages did not translate into superior functional improvement by 1 year. Longer-term standardized comparative studies are required.

## Introduction

Total knee arthroplasty (TKA) is a definitive treatment for end-stage osteoarthritis [1]. It is reliable in providing pain relief and functional restoration for patients with debilitating joint disease [2]. Long-term survivorship of the TKA prosthesis is highly dependent on surgical precision, such as the accuracy of limb alignment, component positioning, and the correct balancing of soft tissues [3–5]. Moreover, knee arthroplasty performed through manual instrumentation is subject to technical limitations and high variability, as surgeon-defined assessments are subjective and prone to inconsistencies [6, 7]. To address this, robotic-arm-assisted TKA (rTKA) has been increasingly adopted to enhance surgical accuracy and minimize outliers [8, 9]. Consequently, robotic platforms enable surgeons to execute real-time adjustments, reducing iatrogenic errors and facilitating specific alignment philosophies [10].

For years, mechanical alignment (MA) has dominated the field, serving as the “gold standard” for TKA [11]. This approach aims to achieve a neutral mechanical axis by performing bone resections perpendicular to the coronal plane, thereby ensuring an even load distribution and implant longevity [12, 13]. However, despite excellent results, a significant subset of patients remains dissatisfied due to residual pain or functional limitations [14]. This dissatisfaction encouraged the development of kinematic alignment (KA), which recreates the patient’s native anatomy without enforcing a standardized neutral alignment [15]. Building on this individualized approach, functional alignment (FA) has emerged as a robot-enabled, patient-specific alignment philosophy in TKA. Rather than representing a single fixed alignment target, FA uses preoperative planning and intraoperative robotic assessment to individualize femoral and tibial component position and sizing in the coronal, sagittal, and axial planes. This approach has been described as a technique that respects individual bony and soft-tissue phenotypes within defined limitations and aims to restore constitutional bony alignment while balancing soft-tissue laxity through implant positioning and sizing [16, 17]. In randomized robotic TKA, FA has also been operationalized by using initial virtual component positioning to match native knee anatomy, followed by adjustments for soft-tissue balance before bone cuts are performed [18]. Therefore, soft-tissue preservation is not the sole definition of FA, but rather a consequence of optimizing bone resections and implant positioning before performing additional ligamentous releases. This is supported by evidence that FA may achieve a balanced TKA more consistently than MA or KA before soft-tissue release [19]. However, FA remains variably defined in the literature and has recently been described as an “umbrella term,” because the desired balance can be reached through multiple combinations of component-position adjustments and soft-tissue strategies [20].

While the current literature features extensive meta-analyses evaluating the MA versus KAs, there remains a gap regarding the head-to-head comparison of robotic-assisted MA and robotic-assisted FA [11, 21]. Consequently, it remains unclear whether robotic FA provides measurable advantages over robotic MA in specific perioperative and clinical outcomes, including the need for soft-tissue release, early and mid-term ROM, PROMs, functional score improvement, and surgical duration. Available comparative studies have reported discordant findings. Several cohorts suggest that FA yields superior Forgotten Joint Scores (FJS-12) and other patient-reported outcome measures (PROMs) [22–25]. These findings are often contradicted by other studies reporting no statistically significant difference in clinical advantage [18]. Furthermore, heterogeneity persists across functional metrics, specifically range of motion (ROM). Reports of improved early ROM in FA groups are frequently offset by other studies demonstrating equivalence in ROM throughout the postoperative period [23–25]. As for surgical duration, while some evidence indicates that the FA technique significantly shortens the procedure, other studies report no significant difference in operative time compared to MA [18, 23, 24, 26]. These persistent discrepancies across multiple clinical studies necessitate a meta-analysis to consolidate findings into a single, evidence-based study.

## Material and methods

### Search strategy

**Figure 1:**
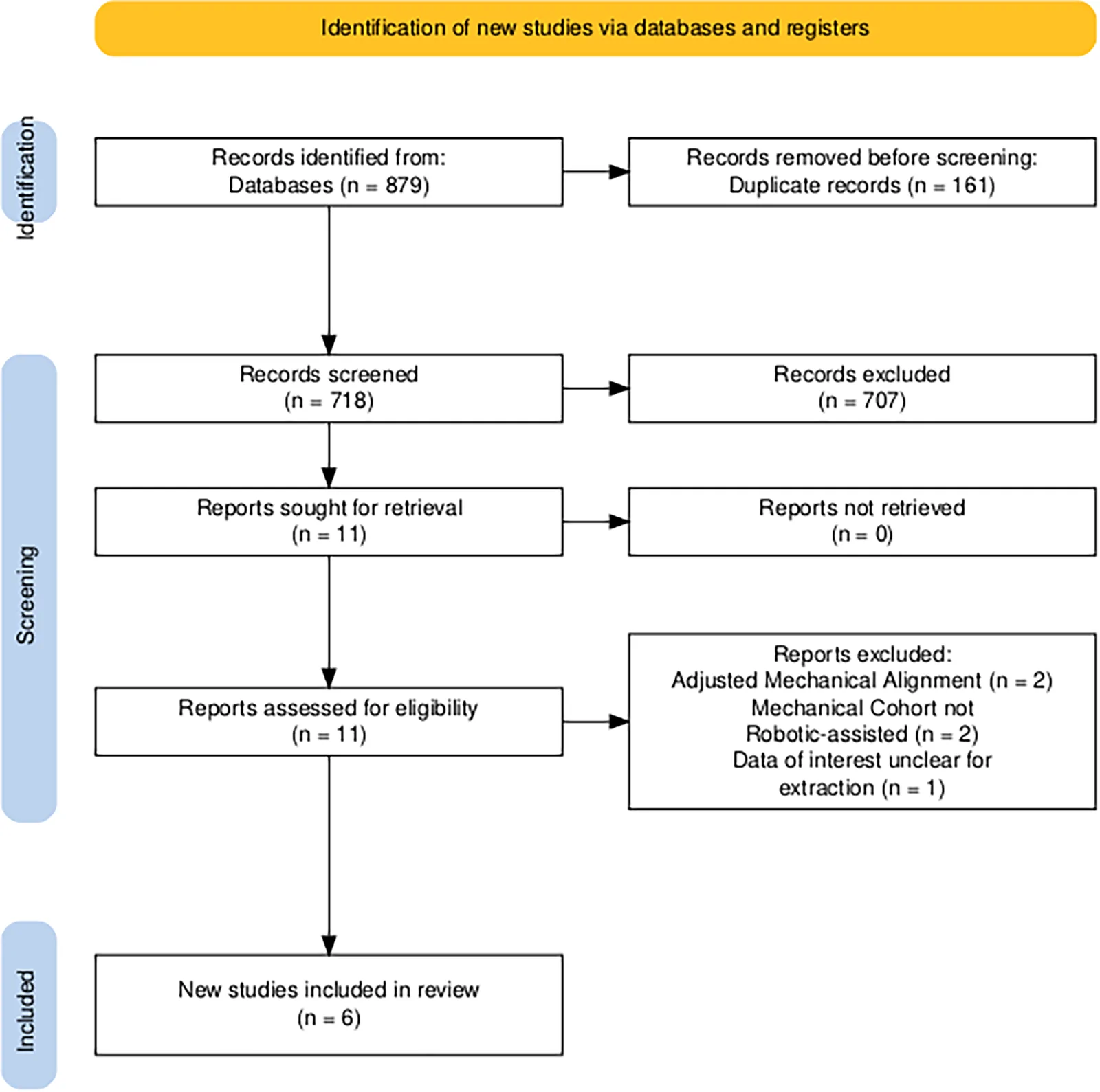
PRISMA flow diagram of the included studies.

This meta-analysis was performed in accordance with the Preferred Reporting Items for Systematic Review and Meta-Analyses (PRISMA) guidelines [27]. No prospective protocol was registered in PROSPERO or another systematic review registry before conducting this meta-analysis. Medline (PubMed) was searched using the following Title/Abstract [TIAB] strategy: ((“knee arthroplasty”[TIAB] OR “knee replacement”[TIAB] OR “TKA”[TIAB] OR “TKR”[TIAB]) AND (“functional alignment”[TIAB]) AND (“mechanical alignment”[TIAB]) AND (“robotic”[TIAB] OR “robot-assisted”[TIAB] OR “robot”[TIAB] OR “robotic arm”[TIAB])). The Cochrane Library was searched via MeSH descriptor “Arthroplasty, Replacement, Knee” combined with free-text terms “functional alignment,” “mechanical alignment,” and “robotic.” Google Scholar (pages 1–20) was searched using the phrase: “functional alignment compared to mechanical alignment in robotic knee arthroplasty.” Reference lists of all included studies were additionally hand-searched. The process is summarized in the PRISMA flowchart (Figure 1).

### Eligibility criteria

Studies were eligible for inclusion irrespective of publication language if they compared FA versus MA in adult patients (18 years or older) undergoing primary rTKA. Non-comparative studies, conference abstracts, study protocols, and review articles were excluded from the analysis. Furthermore, studies were excluded if they involved revision surgery or if data of interest were not extractable.

Two separate reviewers independently determined the eligibility of the included studies. Disagreements, if present, were discussed and resolved by a third senior author.

### Data extraction

Data were extracted and tabulated using a Microsoft Excel spreadsheet (version 2021; Microsoft). Collected data included PROMs, ROM, surgical duration, incidence of soft-tissue release, and study characteristics such as country of origin, study design, robotic system used, latest follow-up time, and patients’ demographics. PROMs included the Western Ontario and McMaster Universities Osteoarthritis Index (WOMAC) (scored 0–96, with 96 representing the worst health status), the Oxford Knee Score (OKS) (scored 0–48, with 0 representing the worst outcomes), and the FJS (scored 0–100, with 0 representing the worst outcome).

Knee motion outcomes were extracted as change from baseline at each postoperative time point. In Chompoosang et al. [23] the reported flexion range was extracted. In Young et al. [18], although both extension and flexion were reported separately, flexion was extracted to maintain consistency with Chompoosang et al. [23] and with studies reporting non-separated ROM, and to avoid deriving total-arc SDs using additional correlation assumptions. In Naito et al. [25] and Wang et al. [24], which reported a single ROM value without separate flexion and extension components, the reported ROM values were extracted as presented. Accordingly, this outcome should be interpreted as change in reported knee motion, that is, ROM or flexion range, rather than uniformly recalculated total arc of motion.

WOMAC scores were converted to the OKS using previously reported techniques [28]. In our data set, OKS and converted WOMAC scores were represented as functional scores following their conversion.

Minimum clinically important differences (MCIDs) were utilized to evaluate the clinical relevance of the pooled results beyond statistical significance. An MCID of 5 points was utilized for the OKS as established in the literature [21, 29]. For the FJS-12, the threshold for a clinically meaningful improvement was defined as 13.7–14 points [21, 30]. Regarding ROM, a clinically important change in flexion was defined as falling between 3.8° and 6.4°, but in patients with knee osteoarthritis after non-surgical interventions [21, 31].

### Risk of bias assessment

Two authors independently assessed the risk of bias of the non-randomized studies using the Risk Of Bias In Nonrandomized Studies-of Interventions (ROBINS-I) tool [32], whereas the revised RoB-2 tool was used to evaluate the risk of bias of the randomized studies [33]. Any disagreement, if present, was discussed and resolved by a third independent senior author.

### Data analyses

Statistical analysis for this review was performed utilizing Review Manager (RevMan) 5.4 software. Continuous outcomes, including FJS, OKS, converted WOMAC, and ROM, were evaluated using mean differences (MDs) with associated 95% confidence intervals (CIs). Categorical data, specifically the incidence of soft-tissue releases, were analyzed using odds ratios (ORs). Q tests and I^2^ statistics evaluated heterogeneity. If considerable heterogeneity was indicated by *p* ≤ 0.05 or I^2^ > 50%, a random-effects model was used. Otherwise, the fixed-effect model was implemented. A statistically significant result is shown by *p* ≤ 0.05.

Of note, the mean ± Standard deviation change in scores from pre- to postoperative specific follow-up points was calculated and used for the pooled analysis to minimize the impact of baseline heterogeneity and enhance clinical interpretability of postoperative improvement. Therefore, changes from baseline values were used for all continuous data, except FJS scores at 3 and 6 months.

## Results

### Characteristics of the included studies

**Figure 2:**
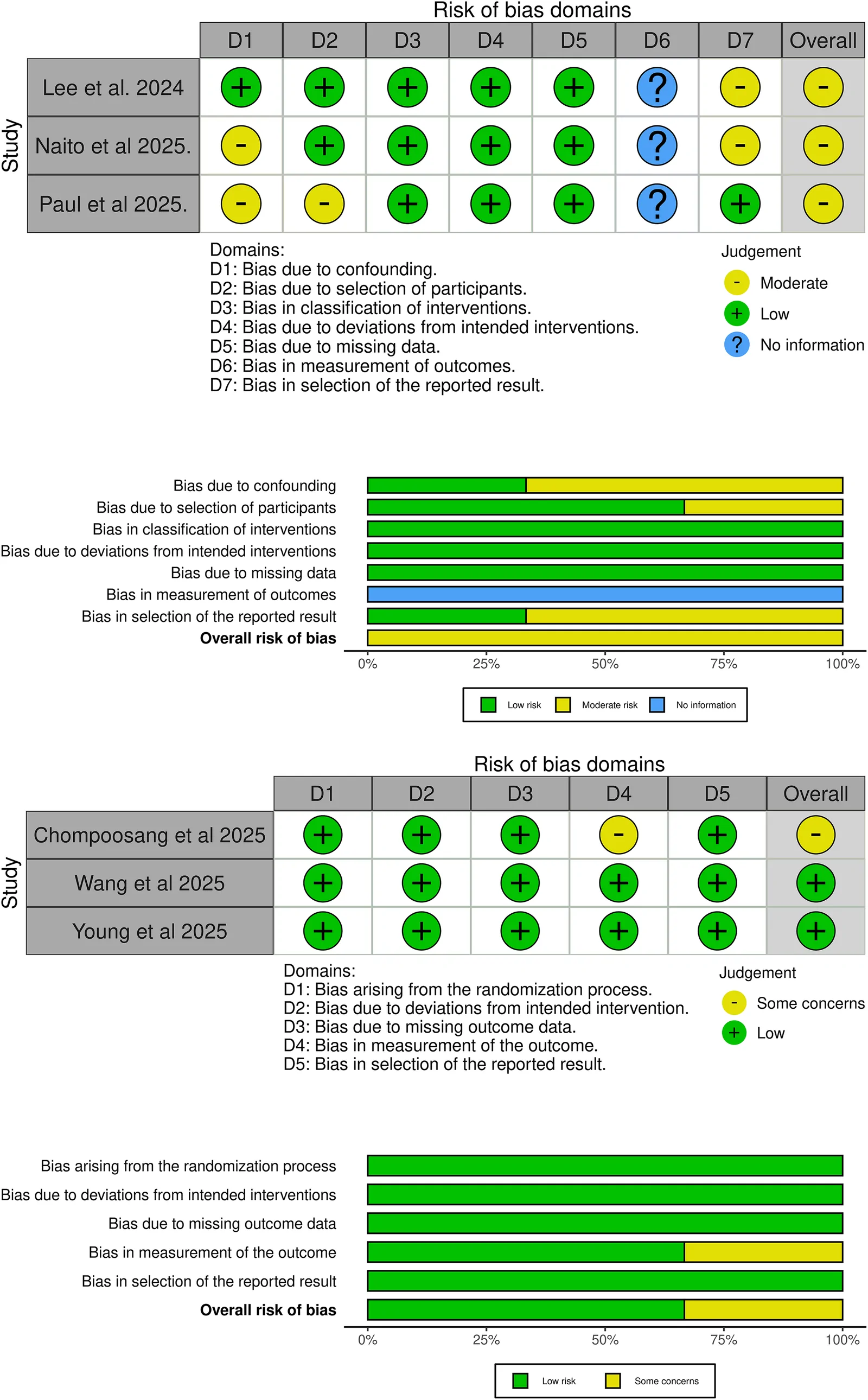
(A): Quality assessment of the included studies using the ROBINS-I tool. (B): Quality assessment of the included studies using the RoB-2 tool.

Six studies met the inclusion criteria [18, 22–26]. These studies included 645 knees, with 321 in the MA group and 324 in the FA group. Study characteristics are summarized in Table 1. The results of the risk-of-bias assessment of the included studies are summarized in Figures 2A and 2B.

**Table 1:** Study characteristics.

Study	Country	Design	Robotic system	Latest follow-up	Cohort size (FA/MA)	Gender (M/W)*	Mean age (Study)
Chompoosang (2025) [23]	Thailand	RCT	Mako	6 months	30 / 30	4 / 26	67.9 yrs
Lee (2024) [22]	South Korea	Retrospective	Mako	1 year	70 / 70	33 / 107	69.7 yrs
Naito (2025) [25]	Japan	Retrospective	ROSA	6 months	31 / 31	15 / 47	73.9 yrs
Paul (2026) [26]	India	Prospective non-randomized	Mako	18 months	20 / 20	34 / 6	64.4 yrs
Wang (2025) [24]	China	RCT	Mako	6 months	50 / 49	18 / 81	67.5 yrs
Young (2025) [18]	New Zealand	RCT	Mako	2 years	123 / 121	128 / 116	67.0 yrs

*Gender distribution represents patient level counts. In Chompoosang et al., 30 patients underwent bilateral TKA, with each patient contributing one FA knee and one MA knee. Therefore, the cohort size is presented as 30/30 knees, whereas gender is presented for the 30 unique patients (4 male/26 female).

### Incidence of patients needing soft-tissue release

**Figure 3:**
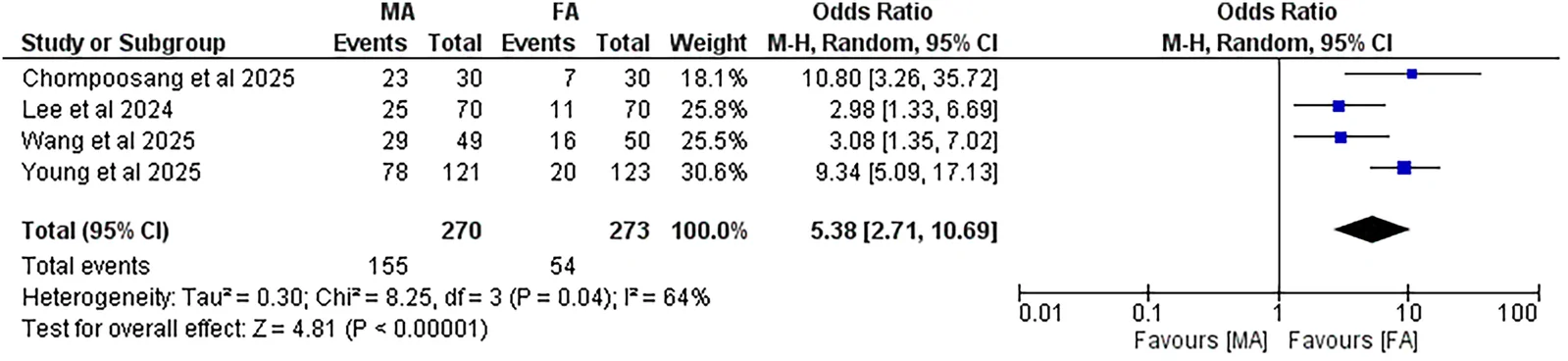
Forest plot showing the difference in the rate of additional soft-tissue releases.

Data from four studies involving 543 patients reported the incidence of patients needing soft-tissue release. The MA group had significantly higher odds of requiring any additional intraoperative soft-tissue release compared with the FA group (OR = 5.38; 95% CI: 2.71–10.69; *P* < 0.00001, Figure 3), indicating that MA patients were approximately 5.4 times more likely to require at least one release than FA patients.

### ROM

**Figure 4:**
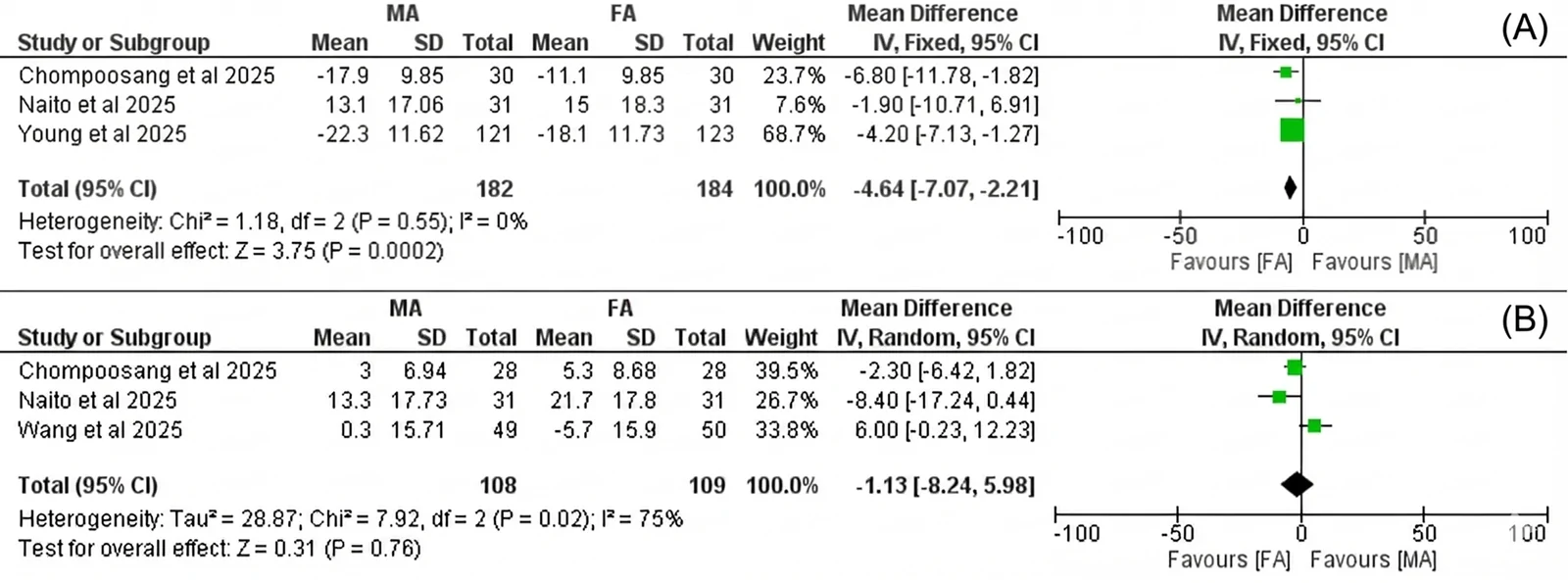
(A): Forest plot showing the difference in ROM improvement at ≤6 weeks. (B): Forest plot showing the difference in ROM improvement at 6 months.

Three studies, including 366 patients, reported ROM at ≤6 weeks. The FA group achieved a significantly greater ROM improvement during this early recovery period (MD = −4.64°; 95% CI: −7.07 to −2.21, *P* = 0.0002; Figure 4A). This 4.64° difference correlated with clinical significance, as it falls within the established MCID range for knee flexion (3.8°–6.4°).

At the 6-month follow-up (3 studies, 217 patients), the difference in ROM was no longer statistically significant (MD = −1.13°; 95% CI: −8.24 to 5.98, *P* = 0.76; Figure 4B).

### PROMs

**Figure 5:**
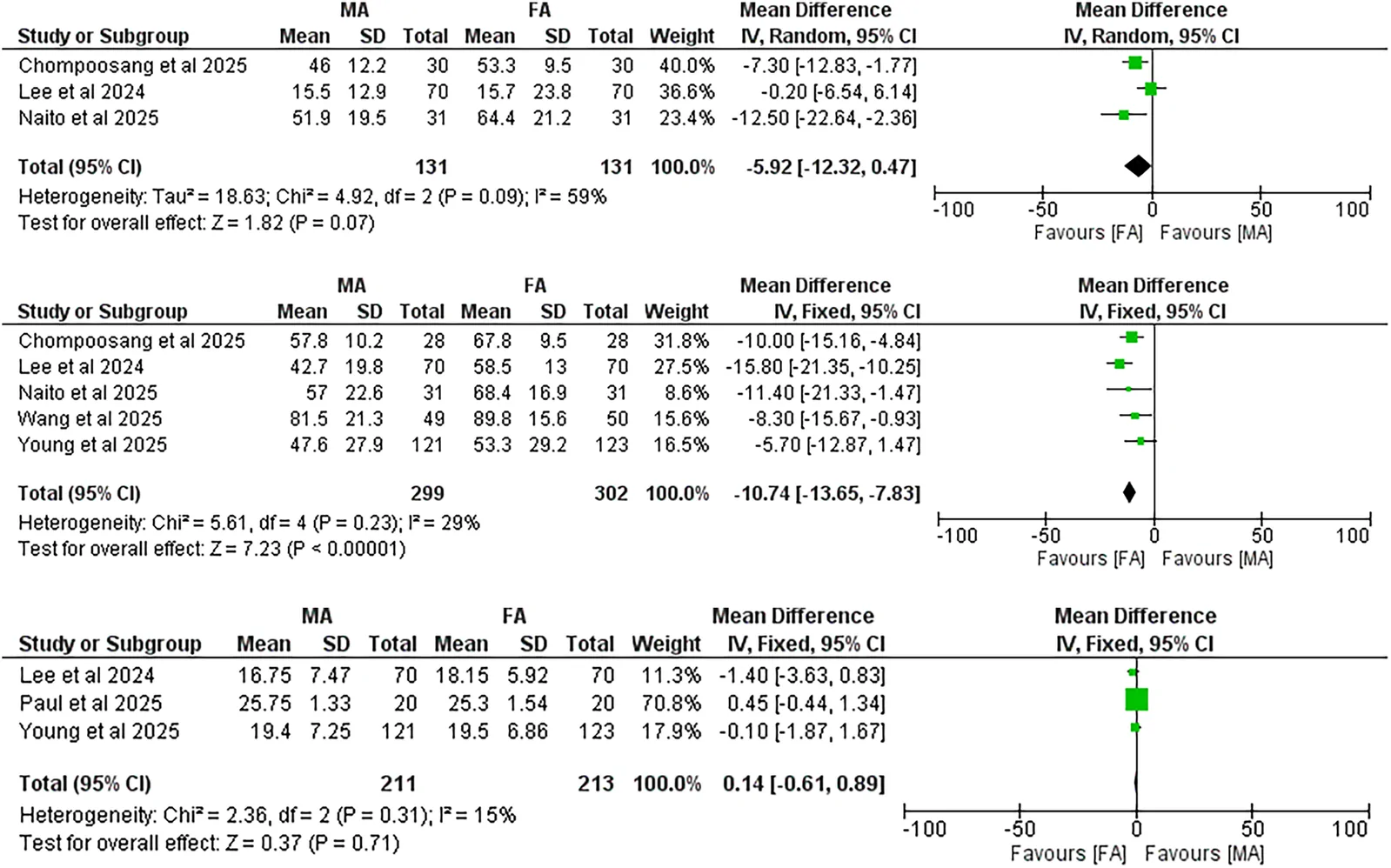
(A): Forest plot showing the difference in FJS scores at 3 months post-operatively. (B): Forest plot showing the difference in FJS scores at 6 months post-operatively. (C) Forest plot showing the difference in functional scores improvement at 1 year post-operatively.

Three studies, including 262 patients, reported data regarding the FJS-12 at 3 months postoperatively. The difference between the two groups was not statistically significant (MD = −5.92; 95% CI: −12.32 to 0.47, *P* = 0.07; Figure 5A).

Five studies, involving 601 patients, reported the FJS-12 at 6 months. The FA group demonstrated significantly better FJS-12 scores compared to the MA group (MD = −10.74; 95% CI: −13.65 to −7.83, *P* < 0.00001; Figure 5B). However, while statistically significant, this 10.74-point improvement did not reach the MCID threshold of 13.7 points.

Regarding the functional score improvement at 1 year, data from three studies (424 patients) showed no significant difference between alignment strategies (MD = 0.14; 95% CI: −0.61 to 0.89, *P* = 0.71; Figure 5C).

### Surgical duration

**Figure 6:**
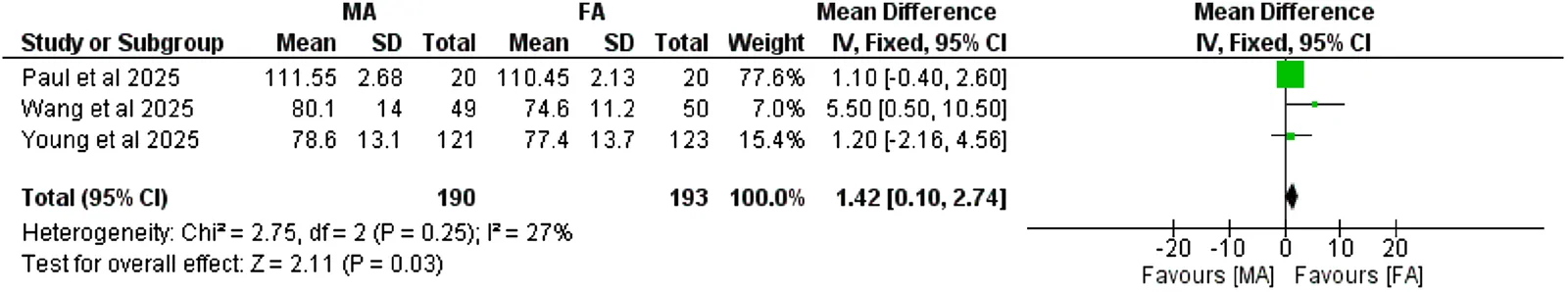
Forest plot showing the difference in the surgical duration.

Three studies, involving 383 patients, provided data on surgical duration. The operative time was significantly shorter in the FA group compared to the MA group (MD = 1.42; 95% CI: 0.10 to 2.74, *P* = 0.03, Figure 6)

## Discussion

This meta-analysis compared robotic-assisted FA and MA in primary TKA across six comparative studies including 645 knees. The principal finding was that robotic FA was associated with a significantly lower need for additional soft-tissue release and greater early ROM improvement at ≤6 weeks compared with robotic MA. However, the early ROM advantage was no longer significant by 6 months. FA also demonstrated statistically superior FJS-12 scores at 6 months, but this difference did not reach the established MCID threshold, and pooled functional score improvement at 1 year was comparable between alignment strategies. Surgical duration was statistically shorter with FA, although the magnitude of this difference was small and unlikely to be clinically meaningful in isolation. Taken together, these findings suggest that robotic FA may provide early perioperative and recovery advantages, particularly through reduced soft-tissue release and improved early motion, but current comparative evidence does not demonstrate a clinically meaningful superiority in patient-reported functional outcomes by 1 year. These results are clinically relevant because they help frame robotic FA not as a definitively superior alignment philosophy, but as a patient-specific strategy that may accelerate early recovery while requiring longer-term comparative data to determine whether early advantages translate into durable functional benefit, implant survivorship, or reduced revision risk.

Despite MA TKA’s established success, only 82%–89% of patients who have undergone the operation report satisfaction [34, 35]. This persistent dissatisfaction led to increased interest in FA TKA, which respects constitutional limb anatomy while achieving balanced soft tissues within defined safety limits [16]. And, while rTKA can improve the accuracy and consistency of component placement, its broader impact on surgical efficiency and outcomes remains uncertain [36, 37]. Therefore, this meta-analysis was conducted to compare rTKA with FA or MA across six comparative studies whose results are in discordance [18, 22–26]. The pooled results indicate that FA’s primary benefits are notably reducing the need for soft-tissue releases while achieving superior early ROM improvement. However, these gains appear transient; ROM advantages were lost by 6 months, and statistical improvements in mid-term FJSs failed to reach clinically meaningful thresholds. Moreover, pooled 1-year data showed no significant difference in functional scores between the groups.

This meta-analysis reported a marked reduction in soft-tissue releases with FA, as MA showed approximately 5.4-times higher odds of requiring a release. This lower rate of soft-tissue release with FA may reflect a fundamental difference in how balance is achieved in robotic FA versus MA. In MA, femoral and tibial component positioning is traditionally referenced to the mechanical axes, with bone cuts performed perpendicular to these axes to restore a neutral limb alignment [38]. When residual imbalance persists, soft-tissue releases may then be required to obtain balanced flexion and extension gaps and ligament stability [39]. In contrast, FA attempts to obtain balance earlier in the workflow by adapting component position, sizing, joint-line orientation, femoral rotation, tibial slope, and flexion-extension gaps within predefined safety limits [16, 17]. Therefore, the observed reduction in releases should not be interpreted simply as “soft-tissue preservation,” but rather as the result of shifting part of the balancing process from ligament release to three-dimensional implant planning. This interpretation is supported by evidence that FA can achieve a balanced TKA before soft-tissue release more consistently than MA or KA [19].

FA also demonstrated greater early postoperative improvement in ROM. The pooled difference in ROM improvement at ≤6 weeks (4.64°) is consistent with published estimates for the MCID in knee flexion (3.8°–6.4°) reported in patients with knee osteoarthritis after non-surgical interventions [31]. This could support the plausibility that patients may perceive a meaningful difference during early rehabilitation; however, TKA-specific ROM MCID thresholds remain poorly defined and warrant further investigation. The absence of a significant difference in ROM improvement by 6 months is clinically plausible, as resolution of postoperative inflammation and progression of structured rehabilitation may lead to convergence in motion outcomes across alignment strategies, despite early ROM differences.

PROMs showed a time-dependent pattern. The pooled FJS-12 scores showed a clear advantage for FA at 6 months (MD = −10.74) but did not differ at 3 months. As a measure of joint awareness, the FJS-12 is uniquely sensitive to subtle residual symptoms like stiffness or “unnatural” feel that emerge as patients increase their daily activity. However, clinical relevance remains uncertain because the 10.74-point difference at 6 months fails to meet the established 13.7-point MCID threshold, suggesting these gains may not be fully perceivable by patients [30]. Longer-term comparative FA-versus-MA studies are needed to determine whether the FJS-12 difference observed in early follow-up persists over time and whether it reaches clinically meaningful thresholds. This need for longer-term comparative evidence should be distinguished from the existing short- to mid-term literature on FA itself. The present meta-analysis specifically evaluates direct robotic FA-versus-MA comparisons, whereas several non-comparative or indirectly comparative studies have reported encouraging outcomes after robotic-assisted TKA performed according to FA principles. A systematic review reported favorable functional outcomes and minimal early revision risk in robotic FA TKA at a minimum 2-year follow-up, although the authors also highlighted heterogeneity and uncertainty regarding long-term safety [40]. Similarly, recent robotic FA cohort studies have reported comparable functional outcomes, complication rates, and revision rates across different patient subgroups and coronal morphotypes [41, 42]. Therefore, the current evidence gap should be framed not as an absence of short- to mid-term FA data, but as a lack of robust, standardized, long-term comparative trials directly testing robotic FA against robotic MA.

At 1-year postoperatively, pooled analysis of improvement in functional score derived from OKS and WOMAC harmonized to a common scale showed no statistically significant difference between FA and MA cohorts. This may indicate that early clinical advantages associated with FA likely reflect an accelerated recovery trajectory, rather than a higher functional level at extended follow-up. Furthermore, the absence of a statistical difference in improvement of functional scores at 12 months may be attributable to the inherent measurement limitations of conventional PROMs. As the OKS often exhibits ceiling effects in TKA cohorts [29, 43], which makes it harder to detect modest differences in alignment in well-performed robotic TKA. This may be more perceivable by more sensitive tools like the FJS-12 [44, 45].

Finally, surgical duration significantly favored FA by 1.42 min, a difference unlikely to be clinically meaningful in isolation and readily confounded by workflow and surgeon experience. Furthermore, a meta-analysis on rTKA found that operative time improves with surgeon experience [46].

### Strengths and limitations

Several potential limitations exist in this meta-analysis. Soft-tissue release was defined as any intraoperative periarticular release performed to achieve gap balance, reported as the proportion of patients requiring at least one release. However, the specific procedures qualifying as a “release” differed across studies. This heterogeneity in release definition is to be acknowledged as a limitation and contributes to the observed between-study variance. In addition, knee motion was inconsistently reported across studies, with some reporting flexion range and others reporting non-separated ROM. Therefore, total arc of motion could not be uniformly recalculated, and this outcome should be interpreted as change in reported knee motion rather than a standardized ROM construct.

Follow-up across included studies was predominantly short-to-midterm (6 months–2 years), restricting conclusions about wear, late instability, and revision risk. Generalizability is further constrained by the robotic platforms used, predominantly a single system, with only one ROSA cohort, and by variability in how FA parameters, limits, and balancing targets were defined. Furthermore, the inclusion of retrospective designs and the robotic learning curve may have introduced inherent bias and heterogeneity. To mitigate this, we implemented random-effects models when necessary and followed strict Cochrane guidelines. Despite these constraints, this is the first meta-analysis specifically comparing robotic FA versus MA, providing an evidence-based framework for personalized surgical strategies.

## Conclusion

Robotic-assisted FA, when compared with MA, reduces the necessity for soft-tissue releases and accelerates early recovery through improved ROM. And while FA demonstrates statistically superior joint awareness at 6 months postoperatively, these gains do not currently meet established thresholds for clinical importance. By 1 year, functional scores are not different, though subtle advantages of FA may be hidden by the ceiling effects inherent in standard PROM scales. Therefore, while FA offers an earlier functional recovery, larger trials with extended follow-up, incorporating more sensitive outcome scales, are essential to confirm its long-term impact on patient outcomes, implant durability, and revision risk.

## Abbreviations

TKA: total knee arthroplasty
FA: functional alignment
MA: mechanical alignment
rTKA: robotic-assisted total knee arthroplasty
ROM: range of motion
PROMs: patient-reported outcome measures
FJS-12: Forgotten Joint Score-12
OKS: Oxford Knee Score
WOMAC: Western Ontario and McMaster Universities Osteoarthritis Index
MCID: minimum clinically important difference
PRISMA: Preferred Reporting Items for Systematic Reviews and Meta-Analyses
ROBINS-I: Risk Of Bias In Non-randomized Studies of Interventions
RoB-2: revised Cochrane risk-of-bias tool for randomized trials

## Data Availability

All data analyzed in this study are derived from previously published studies cited in the reference list. Extracted data supporting the findings of this study are available from the corresponding author upon reasonable request.

## References

[1] Skou ST, Roos EM, Laursen MB, et al. (2015) A randomized, controlled trial of total knee replacement. N Engl J Med 373(17), 1597–1606. 26488691 10.1056/NEJMoa1505467

[2] Carr AJ, Robertsson O, Graves S, et al. (2012) Knee replacement. Lancet 379(9823), 1331–1340. 22398175 10.1016/S0140-6736(11)60752-6

[3] Karuppal R (2016) Kinematic alignment in total knee arthroplasty: Does it really matter? J Orthop 13(4), A1–A3. 10.1016/j.jor.2016.10.001 PMC5103048 27857485

[4] Sharkey PF, Lichstein PM, Shen C, et al. (2014) Why are total knee arthroplasties failing today-has anything changed after 10 years? J Arthroplasty 29(9), 1774–1778. 25007726 10.1016/j.arth.2013.07.024

[5] Gromov K, Korchi M, Thomsen MG, et al. (2014) What is the optimal alignment of the tibial and femoral components in knee arthroplasty? Acta Orthop 85(5), 480–487. 25036719 10.3109/17453674.2014.940573 PMC4164865

[6] Orsi AD, Coffey S, Slotkin E, et al. (2025) Soft tissue balance profiles differ between manual and robotically assessed gaps in total knee arthroplasty. Int J Med Robot 21(6), e70124. 41318912 10.1002/rcs.70124

[7] Tran JYS, Lee JWL, Wong CK, et al. (2025) A comparison on the clinical outcomes of using intraoperative load sensors versus manual balancing in total knee arthroplasty: a systematic review and meta-analysis. J Orthop Surg Res 20(1), 970. 41204363 10.1186/s13018-025-06394-8 PMC12595699

[8] García-Sanz F, Sosa-Reina MD, Jaén-Crespo G, et al. (2025) Redefining knee arthroplasty: Does robotic assistance improve outcomes beyond alignment? An evidence-based umbrella review. J Clin Med 14(8), 2588. 40283417 10.3390/jcm14082588 PMC12028302

[9] Bourgeault-Gagnon Y, Salmon LJ, Lyons MC (2024) Robotic-assisted total knee arthroplasty improves accuracy and reproducibility of the polyethylene insert thickness compared to manual instrumentation or navigation: a retrospective cohort study. Arthroplast Today 30, 101489. 39492997 10.1016/j.artd.2024.101489 PMC11530840

[10] Wong WK, Abu Bakar Sajak A, Chua HS (2024) Real-world accuracy of robotic-assisted total knee arthroplasty and its impact on expedited recovery. J Robot Surg 18(1), 309. 39105997 10.1007/s11701-024-02059-6 PMC11303575

[11] Wang G, Chen L, Luo F, et al. (2024) Superiority of kinematic alignment over mechanical alignment in total knee arthroplasty during medium- to long-term follow-up: a meta-analysis and trial sequential analysis. Knee Surg Sports Traumatol Arthrosc 32(5), 1240–1252. 38488220 10.1002/ksa.12093

[12] Roussot MA, Vles GF, Oussedik S (2020) Clinical outcomes of kinematic alignment versus mechanical alignment in total knee arthroplasty: a systematic review. EFORT Open Rev 5(8), 486–497. 32953134 10.1302/2058-5241.5.190093 PMC7484715

[13] Elmajee M, Mersal M, Ben Nafa W, et al. (2025) Exploring knee alignment: demystifying traditional and emerging approaches. Cureus 17(9), e91423. 41040791 10.7759/cureus.91423 PMC12487995

[14] Lychagin AV, Gritsyuk AA, Elizarov MP, et al. (2025) Mechanical versus restrictive kinematic alignment in robotic-assisted total knee arthroplasty: a randomized controlled trial. Diagnostics (Basel) 15(19), 2524. 41095743 10.3390/diagnostics15192524 PMC12523575

[15] Howell SM, Howell SJ, Kuznik KT, et al. (2013) Does a kinematically aligned total knee arthroplasty restore function without failure regardless of alignment category? Clin Orthop Relat Res 471(3), 1000–1007. 22996362 10.1007/s11999-012-2613-z PMC3563808

[16] Shatrov J, Foissey C, Kafelov M, et al. (2023) Functional alignment philosophy in total knee arthroplasty: rationale and technique for the valgus morphotype using an image-based robotic platform and individualized planning. J Pers Med 13(2), 212. 36836446 10.3390/jpm13020212 PMC9961945

[17] Shatrov J, Batailler C, Sappey-Marinier E, et al. (2022) Functional alignment philosophy in total knee arthroplasty: rationale and technique for the varus morphotype using a CT-based robotic platform and individualized planning. SICOT J 8, 11. 35363136 10.1051/sicotj/2022010 PMC8973302

[18] Young SW, Tay ML, Kawaguchi K, et al. (2025) The John N. Insall Award: functional versus mechanical alignment in total knee arthroplasty: a randomized controlled trial. J Arthroplasty 40(7 Suppl 1), S20–S30.e2. 40023458 10.1016/j.arth.2025.02.065

[19] Clark G, Steer R, Wood D (2023) Functional alignment achieves a more balanced total knee arthroplasty than either mechanical alignment or kinematic alignment prior to soft tissue releases. Knee Surg Sports Traumatol Arthrosc 31(4), 1420–1426. 36116071 10.1007/s00167-022-07156-3 PMC10050049

[20] Klasan A, Jeremic D, Neri T, et al. (2025) Functional alignment in total knee arthroplasty is an umbrella term-a call for better definition and reporting quality! Knee Surg Sports Traumatol Arthrosc 33(12), 4275–4281. 40652378 10.1002/ksa.12774 PMC12684296

[21] Nucci N, Chakrabarti M, DeVries Z, et al. (2025) Kinematic alignment does not result in clinically important improvements after TKA compared with mechanical alignment: a Meta-analysis of randomized trials. Clin Orthop Relat Res 483(6), 1020–1030. 39842026 10.1097/CORR.0000000000003356 PMC12106242

[22] Lee JH, Kwon SC, Hwang JH, et al. (2024) Functional alignment maximises advantages of robotic arm-assisted total knee arthroplasty with better patient-reported outcomes compared to mechanical alignment. Knee Surg Sports Traumatol Arthrosc 32(4), 896–906. 38454836 10.1002/ksa.12120

[23] Chompoosang T, Ketkaewsuwan U, Ploynumpon P (2025) Comparative effects of mechanical and functional alignment in bilateral robotic total knee arthroplasty: a randomized controlled trial. Arthroplasty 7(1), 25. 40329420 10.1186/s42836-025-00310-5 PMC12057096

[24] Wang Q, Yan Z, Wang Q, et al. (2025) The early outcome of robotic-assisted functional versus mechanical alignment in total knee arthroplasty: a prospective randomized control trial. J Arthroplasty 40(11 Suppl 1), S36–S44. 10.1016/j.arth.2025.08.030 40840662

[25] Naito Y, Tone S, Kobayashi G, et al. (2025) Comparison of clinical outcomes and soft tissue laxity between robot-assisted functionally and mechanically aligned total knee arthroplasty. Cureus 17(8), e91084. 40895666 10.7759/cureus.91084 PMC12391744

[26] Paul AA, Thilak J (2026) A prospective study to compare clinical and radiological outcomes of functionally versus mechanically aligned knees following robotic total knee arthroplasty. J Knee Surg 39(5), 234–241. 41027465 10.1055/a-2712-4085

[27] Page MJ, McKenzie JE, Bossuyt PM, et al. (2021) The PRISMA 2020 statement: an updated guideline for reporting systematic reviews. Syst Rev 10(1), 89. 33781348 10.1186/s13643-021-01626-4 PMC8008539

[28] Murad MH, Wang Z, Chu H, et al. (2019) When continuous outcomes are measured using different scales: guide for meta-analysis and interpretation. BMJ 364, k4817. 30670455 10.1136/bmj.k4817 PMC6890471

[29] Clement ND, MacDonald D, Simpson AHRW (2014) The minimal clinically important difference in the Oxford Knee Score and Short Form 12 score after total knee arthroplasty. Knee Surg Sports Traumatol Arthrosc 22(8), 1933–1939. 24253376 10.1007/s00167-013-2776-5

[30] Clement ND, Scott CEH, Hamilton DF, et al. (2021) Meaningful values in the Forgotten Joint Score after total knee arthroplasty. Bone Joint J 103-B(5), 846–854. 33934639 10.1302/0301-620X.103B5.BJJ-2020-0396.R1

[31] Silva MDC, Woodward AP, Fearon AM, et al. (2024) Minimal clinically important change of knee flexion in people with knee osteoarthritis after non-surgical interventions using a meta-analytical approach. Syst Rev 13(1), 50. 38303000 10.1186/s13643-023-02393-0 PMC10832130

[32] Sterne JA, Hernán MA, Reeves BC, et al. (2016) ROBINS-I: a tool for assessing risk of bias in non-randomised studies of interventions. BMJ 355, i4919. 27733354 10.1136/bmj.i4919 PMC5062054

[33] Sterne JAC, Savović J, Page MJ, et al. (2019) RoB 2: a revised tool for assessing risk of bias in randomised trials. BMJ 366, l4898. 31462531 10.1136/bmj.l4898

[34] Parratte S, Pagnano MW, Trousdale RT, et al. (2010) Effect of postoperative mechanical axis alignment on the fifteen-year survival of modern, cemented total knee replacements. J Bone Joint Surg Am 92(12), 2143–2149. 20844155 10.2106/JBJS.I.01398

[35] Bourne RB, Chesworth BM, Davis AM, et al. (2010) Patient satisfaction after total knee arthroplasty: Who is satisfied and who is not? Clin Orthop Relat Res 468(1), 57–63. 19844772 10.1007/s11999-009-1119-9 PMC2795819

[36] Kort N, Stirling P, Pilot P, et al. (2022) Robot-assisted knee arthroplasty improves component positioning and alignment, but results are inconclusive on whether it improves clinical scores or reduces complications and revisions: a systematic overview of Meta-analyses. Knee Surg Sports Traumatol Arthrosc 30(8), 2639–2653. 33666686 10.1007/s00167-021-06472-4

[37] Deckey DG, Rosenow CS, Verhey JT, et al. (2021) Robotic-assisted total knee arthroplasty improves accuracy and precision compared to conventional techniques. Bone Joint J 103-B(6 Suppl A), 74–80. 34053292 10.1302/0301-620X.103B6.BJJ-2020-2003.R1

[38] Cherian JJ, Kapadia BH, Banerjee S, et al. (2014) Mechanical, anatomical, and kinematic axis in TKA: concepts and practical applications. Curr Rev Musculoskelet Med 7(2), 89–95. 24671469 10.1007/s12178-014-9218-y PMC4092202

[39] Meloni MC, Hoedemaeker RW, Violante B, et al. (2014) Soft tissue balancing in total knee arthroplasty. Joints 2(1), 37–40. 25606540 PMC4295665

[40] Giovanoulis V, Vasiliadis AV, Andriollo L, et al. (2025) Functional alignment in robotic total knee arthroplasty provides favourable outcomes and minimal early revisions: a systematic review and meta-analysis. Knee Surg Sports Traumatol Arthrosc, 1–15. 10.1002/ksa.70226. PMC13502434 41358691

[41] Koutserimpas C, Andriollo L, Gregori P, et al. (2025) Robotic total knee arthroplasty with functional alignment yields comparable outcomes across age and gender groups. J ISAKOS 14, 100930. 40716713 10.1016/j.jisako.2025.100930

[42] Koutserimpas C, Caria C, Gregori P, et al. (2025) Functional alignment in robotic total knee arthroplasty achieves comparable outcomes in varus and valgus knees despite distinct intraoperative strategies: analysis of 355 consecutive cases. Knee Surg Sports Traumatol Arthrosc 33(11), 3925–3934. 40621947 10.1002/ksa.12764 PMC12582244

[43] Jenny JY, Louis P, Diesinger Y (2014) High Activity Arthroplasty Score has a lower ceiling effect than standard scores after knee arthroplasty. J Arthroplasty 29(4), 719–721. 23953961 10.1016/j.arth.2013.07.015

[44] Adriani M, Malahias MA, Gu A, et al. (2020) Determining the validity, reliability, and utility of the Forgotten Joint Score: a systematic review. J Arthroplasty 35(4), 1137–1144. 31806559 10.1016/j.arth.2019.10.058

[45] Giesinger K, Hamilton DF, Jost B, et al. (2014) Comparative responsiveness of outcome measures for total knee arthroplasty. Osteoarthritis Cartilage 22(2), 184–189. 24262431 10.1016/j.joca.2013.11.001 PMC3988962

[46] Abdel Khalik H, Abesteh J, Aldawodi M, et al. (2025) The learning curve of robotic-assisted total knee arthroplasty: a systematic review and meta-analysis. J Robot Surg 19(1), 456. 40768113 10.1007/s11701-025-02576-y

